# Factors influencing vaccine acceptance and hesitancy in three informal settlements in Lusaka, Zambia

**DOI:** 10.1016/j.vaccine.2018.07.042

**Published:** 2018-09-05

**Authors:** Miguel Pugliese-Garcia, Leonard W. Heyerdahl, Chanda Mwamba, Sharon Nkwemu, Roma Chilengi, Rachel Demolis, Elise Guillermet, Anjali Sharma

**Affiliations:** a Centre for Infectious Disease Research in Zambia (CIDRZ), Plot # 34620, P.O. Box 34681, Lusaka 10101, Zambia; b Agence de Médecine Préventive, Abidjan, Cote d’Ivoire

**Keywords:** Vaccine hesitancy, Vaccine acceptance, Perceived safety, Perceived effectiveness, Informal settlements, Zambia

## Abstract

**Introduction:**

Heterogeneous coverage threatens to compromise the effectiveness of immunization programs in Zambia. Demand-creation initiatives are needed to address this; however, there is incomplete understanding of why vaccine coverage is suboptimal. We investigated overarching perceptions on vaccine acceptability, hesitancy, and accessibility at three informal settlements in Lusaka, Zambia.

**Methods:**

Nested within a cholera vaccination uptake study, we sought to understand overarching perceptions on vaccines’ hesitancy in three informal settlements in Lusaka, Zambia. We conducted 48 focus group discussions with a convenience sample of laypersons, lay healthcare workers, neighbourhood health committee members and vaccinators.

**Results:**

Both laypersons and community-based health actors reported high vaccine acceptance though several sources of hesitancy were reported. Traditional remedies, alcohol use and religious beliefs emerged as drivers of vaccine hesitancy, likely reinforced by a background of distrust towards western medicine. Also mentioned were previous adverse events, fear of injections and low perceived need for immunization. Limited understanding of how vaccines work and overlapping local terms for vaccine and other medical concepts created confusion and inaccurate views and expectations. Some reported refusing injections to avoid pain and perceived risk of infection. Discussants emphasised the importance of education and preferred mobile immunization campaigns, with weekend to reach those with poor access and delivered by a combination of professional and volunteer workers.

**Conclusions:**

Vaccine hesitancy in Zambia is underpinned by many factors including personal experiences with vaccinations, alternative belief models, limited knowledge, deep misunderstanding about how vaccines work, and barriers to access. To overcome these, community-driven models that incorporate factual communication by professionals and operate outside of traditional hours, may help. Better research to understand community preferences for vaccine uptake could inform interventions to improve immunization coverage in Zambia.

## Highlights

• Participants generally expressed acceptance for vaccines but described hesitant individuals.
• Alcohol, prayer and traditional remedies were alternatives reported in the community.
• Adverse effects combined with limited information were likely fostering vaccine hesitancy.
• Limited understanding and misconceptions about vaccines were common.
• Discussants supported delivery strategies that improved education and access.

## 1 Introduction

Despite universal provision, evidence suggests relatively low vaccination coverage in Zambia [1], [2]. For example, though Zambia’s immunisation programme has routinely delivered vaccines for free to infants in all public health facilities since the 1970s, in 2013–2014, less than 60% of children had received the recommended vaccinations by 12 months of age. Coverage varied between vaccines and between doses for a given vaccine, with higher uptake among infants of more educated mothers, urban residents and wealthier households [1]. Lower vaccine coverage in some sub-populations may be due to limited availability of vaccines or vaccination hesitancy (i.e., reluctance) [3], [4]. Under the World Health Organization (WHO) Strategic Advisory Group of Experts' definition, vaccine hesitancy refers to the delay or refusal of vaccination despite its availability [4]. It can be influenced by lack of confidence in recommended vaccines and providers, complacency regarding the need for vaccination, and the perception of how conveniently can be obtained [4]. All of these are shaped by context (e.g., distance to health services, culture, or history) as well as individual and vaccine-specific factors (e.g. perceptions often vary by vaccine) [4], [5], [6], [7] making it important to undertake studies that closely examine people's knowledge and beliefs regarding recommended vaccinations.

Evidence on determinants of vaccine hesitancy in Zambia is scarce. A cross-sectional study on anticipated response to the introduction of the human papillomavirus (HPV) vaccine reported high acceptance among women [8] in contrast, a qualitative paper voiced healthcare workers’ concerns about the influence of male and elders’ consent, distrust of western medicine and low education as barriers to uptake of the vaccine in urban and peri-urban settings, distance to health services, poverty, low health literacy and perceptions on accessibility negatively influenced adults’ decisions to seek care for their children including for immunisation [10], [11]. Modelling on Zambia’s determinants of vaccination estimated that demand-related determinants (i.e., positive attitudes and norms towards vaccines and increased perceived control on vaccination) contributed strongly to completion of all required doses of a vaccine, while supply-related determinants (supplies and human resources) contributed more to vaccine initiation [12]. These included perceived purpose and effectiveness of vaccines and the personnel delivering them (attitudes), social networks and communication (norms), as well as perceived control over time, cost and availability (self-efficacy). While determinants may vary by disease or vaccine, (e.g., a HPV vaccine may be more fear inducing than influenza vaccine), addressing these requires understanding the general perception of vaccinations within the given context [13]. Very recently, a qualitative study reported that mothers in the capital generally had positive views regarding vaccination, but signalled that lack of knowledge and rumours in the community acted as barriers to vaccinating their children [14]. To the best of our knowledge, no study, qualitative or otherwise, has examined general perceptions on vaccination of the wider community and health actors to holistically understand vaccine acceptance and hesitancy in Zambia.

This qualitative study was nested within a larger study on the uptake of two-dose oral cholera vaccine (OCV) in three informal settlements, locally referred to as “compounds”, in Lusaka. Compounds are informal settlements characterised by crowding, poor housing, inadequate water and sanitation and large transient populations from rural areas [15], [16]. Approximately 1.2 million people living in Lusaka compounds are at risk of vaccine-preventable diseases, which can spread rapidly due to a crowded, unsanitary environment and differential vaccine uptake [10], [17], [18]. During February 2016, a cholera outbreak affected several compounds, with 1054 cases reported [15]. In response, the Zambian Ministry of Health began a reactive one-dose OCV campaign in May 2016 [15], [16] followed by a pre-emptive campaign in December 2016. This provided the opportunity to collect information on communities’ views on vaccines, which could contribute to the explanation of differences in vaccine coverage observed in Zambia.

## 2 Methodology

### 2.1 Study design

We conducted a rapid qualitative assessment that included 48 Focus Group Discussions (FGDs) with residents and community-based health actors –lay healthcare workers (HCWs), vaccinators and neighbourhood health committees (NHCs) (Table 1).

**Table 1:** Sample size for procedures before, during and after the 2nd dose campaign.

Participant type	Number of compounds	Phase	Total FGDs
*Laypersons*			
Not vaccinated (0 doses of OCV)			
Men	3	Before & during/after	6
Women	3	Before & during/after	6
1 dose of OCV			
Men	3	Before & during/after	6
Women	3	Before & during/after	6
2 dose of OCV			
Men	3	During/after only	3
Women	3	During/after only	3

Total laypersons			**30**

*Community-based health actors*			
Lay healthcare workers/community assistants	3	Before & during/after	6
Neighbourhood health committees	3	Before & during/after	6
Vaccinators	3	Before & during/after	6

Total health actors			**18**

Participants were recruited using convenience sampling. Each day, research assistants walked from different delivery posts used during the reactive OCV campaign to the nearest gathering to identify adults who reported taking zero or one doses and who were willing to participate in the assessment. They continued recruiting at the house closest to the gathering, moving in concentric circles until they reached eight to twelve people per FGD. During and after the second-dose campaign, they also recruited those who reported taking two doses. Recruited residents were invited to participate in gender and dose-specific groups scheduled for a specific time at the nearest health care facility. Those willing provided a contact number. No-shows were called and if unavailable or unwilling, replaced by new recruits in the vicinity. Community-based health actors were recruited through the compounds’ clinics (government health facilities). Sisters-in-charge (equivalent to clinic managers in other settings) provided these actors with study related information and those interested were invited to vaccinator, lay HCW or NHC FGDs at the clinic.

FGDs began with participants’ informed consent and were conducted in their preferred language (Bemba, English or Nyanja) and recorded with their permission. They also filled a short anonymous questionnaire regarding socio-economic status and vaccines. Being mindful that discussants’ views may have been influenced by the cholera outbreak and the OCV campaigns, questions regarding vaccines in general were asked separately from those specific to OCV and moderators sought clarification when unsure of the point of reference during the FGD.

### 2.2 Data analysis

Audio recordings in local languages were translated and transcribed verbatim in Microsoft Word and subjected to an iterative process of coding in NVivo QSR™. We used latent content analysis [19], reading transcripts repeatedly to develop a sense of the whole before open coding the data using inductive and deductive reasoning. Meaning units against the codes were compared for similarities and differences to create categories of related codes and sub codes. Themes were then generated by interpreting the codes for their underlying meaning and exported as tables in Microsoft Word© for further synthesis. Themes included general acceptability (including competing practices and beliefs and safety), views on effectiveness and preferences for delivery (See Table 2). As laypersons and health actors communicated the same information, we did not differentiate between the two in our results. Questionnaire data were entered into Excel© and simple descriptive analysis was performed using Stata 14.

**Table 2:** Table of themes with supporting quotes.

Meaning unit	Condensed meaning unit	Code
**Theme:** Acceptability and perceived safety		
**Sub-theme:** Preference for traditional and religious alternatives over vaccines		
“If the tradition does not allow us to go [for vaccination], then we won’t go because we believe in herbs or something else that will take care us”	Some people preferred informal traditional alternatives such as traditional brews, herbs and tattoos	Competing traditional beliefs
“Most vaccines they come in injection form, and a lot of religious affiliations like Islam don’t like piercing their bodies, not even tattoos. And other churches also don’t like to tamper with anything that God has created”	Religious beliefs and actors could interact with the decision to get vaccinated	Competing religious beliefs and actors

**Sub-Theme:** Lack of information, past experience and social interactions fan fears		
“Some people say it’s a way that whites wants to kill us through the vaccine by bringing something that we do not really understand; with little knowledge, when someone says something, the first thing it will just stick to your head”	Modern medicine is perceived as external; not to be trusted Past experiences, social interaction and lack of knowledge influences uptake	Modern Medicine; Trust; Experience; Social Interaction
“They say injections are painful and others get swollen when injected” “If pregnant women vaccinate, they may kill the unborn child or maybe the baby will be born with something”	Vaccines are painful, potentially injurious and possibly causing infection	Fear of side-effects Fear for pregnancy

**Theme:** Misconception and perceived effectiveness		
**Sub-Theme:** Vaccines are perceived to be effective		
“The same injections that they give her, even if there are two, they do protect the child from other diseases”	Vaccines protect from diseases	Vaccines protect against all illnesses

**Sub-Theme:** Name, mechanism, purpose and duration of cover differs by vaccines and is not well understood		
“With injections, it goes straight in the blood. It is faster than the oral one”	Injections more effective than oral vaccines	Injections
*“I: What vaccine is available for children?*	Laypersons confuse other medical products with vaccines	Knowledge purpose/protection period
Dewormer … to finish worms in the stomach. Then they are given Vitamin A for their eyes for proper vision”		

**Theme:** Preferences for Vaccine Delivery		
**Sub-Theme:** There is desire for more, and more accurate information from trusted sources		
“We need people to educate us know to about the vaccines, what the vaccines do”	Refusal of vaccines connected to lack of information	Education
“The health worker has more knowledge but the volunteer will also help because he is familiar with the people”	Volunteers understand and care for and are accepted by the community	Preference for volunteers

**Sub-Theme:** People want better access to vaccinations		
“Mobile clinics are very good because other people can’t manage to move long distances” “Most of them prefer routine because … those who work … don’t know which day they would be given permission to attend”	People concerned with access for those far away, old, differently abled, working. Routine campaigns fit people’s needs better	Mode of delivery, mobile; routine Day of provision

### 2.3 Ethical considerations

The University of Zambia Biomedical Research Ethic Committee (UNZABREC) and the National Health Research Authority (NHRA) approved this study.

## 3 Results

### 3.1 Respondents’ characteristics

A total of 281 laypersons and 197 community-based health actors participated in the FGDs, distributed evenly between compounds (Table 3). More than half were female (58%) and 20 to 35 years old (56%). The majority of discussants were educated to High School level (56%) or below (29%) and almost all (99%) self-identified as Christian. Almost half (45%) reported receiving zero OCV doses, followed by one (28%) and two (28%) doses. The majority of the participants had children (64%), and most of those with children would agree to vaccinate them (93%).

**Table 3:** Key socio-economic characteristics in the focus group discussions respondents.

Characteristics	Laypersons	Healthcare actors	Total
Gender			
Female	148 (53)	130 (66)	278 (58)
Male	132 (47)	66 (34)	198 (41)

Age range			
18–20	52 (19)	13 (7)	65 (14)
20–35	174 (62)	95 (48)	269 (56)
36–50	36 (13)	51 (26)	87 (18)
51–68	13 (5)	34 (17)	47 (10)

Compound			
Bauleni	91 (32)	62 (31)	153 (32)
Chawama	101 (36)	69 (35)	170 (36)
Kanyama	89 (32)	66 (34)	155 (32)

Education			
Below High School	105 (37)	36 (18)	141 (29)
High School	143 (51)	125 (63)	268 (56)
Above High School	33 (12)	36 (18)	69 (14)

Religion			
Christian	277 (99)	195 (99)	472 (99)
Other	0 (0)	2 (1)	2 (0)

Times vaccinated			
0	106 (38)	107 (54)	213 (45)
1	60 (21)	72 (37)	132 (28)
2	114 (41)	18 (9)	132 (28)

Has children			
No	120 (43)	49 (25)	169 (35)
Yes	161 (57)	147 (75)	308 (64)

Agrees giving the OCV to their children (% of those with children)			
No	8 (5)	8 (5)	16 (5)
Yes	148 (92)	138 (94)	286 (93)
Don’t know	5 (3)	0 (0)	5 (2)

Total participants	281 (59)	197 (41)	478

### 3.2 Acceptability and perceived safety

All discussants expressed high acceptance of vaccines, associating vaccinations with child health and modernity. However, some participants described hesitant individuals in their community due to competing traditional and religious beliefs, distrust towards modern medicine and past personal and community’s experiences with vaccines and adverse events (Table 4).

**Table 4:** Acceptability and perceived safety of vaccines according to laypersons and health actors.

**Theme: Acceptability and perceived safety**		
**Sub-Theme:** Preference for traditional and religious alternatives over vaccines		
1	Man, 0 doses, before campaign	“[Parents] can prevent me from getting vaccines because they will say, “You can’t go for that [vaccine] when there is medicine here; we have such and such medicine.” You will find that any slight problem that I will have, they will call my grandparents to bring medicine, and then they will tattoo me. Eventually, I will just say, “Give me the tattoo because these tattoos are more effective than vaccines.”
2	Vaccinator, after campaign	“Many people were saying [to vaccinators and health workers] that, “*Kachasu* [local brew] is more effective than the medicine you are giving us. From time memorial, whenever [we] suffered from cholera, we just drank *Kachasu*.”
3	Woman, 1 dose, after campaign	“Even the time they brought the cholera vaccines, some [people] were saying that, “I am a Christian, the holy spirit will protect me.”
4	NHC member, after campaign	“Some people tell us that they refuse to take the vaccine because there is an increase of Satanism in our country. It looks like they are coming to us using the same medicine [used by Satanists] … So people are refusing the vaccine because they say it is all Satanism.”

**Sub-Theme:** Lack of information, past experience and social interactions fan fears		
5	Man, 0 doses, before campaign	“Like my friend was saying, a long time ago, the whites hated Africans. So they inject Africans so that they can die and whites can have the mines and other stuff.”
6	Vaccinator, after campaign	“The first thing we think about in my opinion is, “Do the white people want to experiment on us or what?” Because you will find that whatever test comes, they bring it to Africa [to try].”
7	Woman, 2 doses, after campaign	“Others when they hear some rumours from the compound such as, “Aww, that medicine (synonymous with vaccine), I had rash; that medicine, I had diarrhoea, (…),” they will not take that medicine and tell all the children not to go to take that medicine. So it’s the rumours.”
8	NHC member, after campaign	“They see that if they went to get that vaccine maybe it will give them a problem. Like if they went to get polio or measles or rubella vaccine, they will say that, “Ah! when that baby was injected there, he reacted, developed a rash, or became sick.” So when people hear that, they say, “Me! I won’t go because my child will get sick.”
9	Man 1 dose, before campaign	“For the children they fear that they be injected wrongly, so they prefer oral medication. Many they will not even reach the hospital for fear of an injection.“
10	Lay HCW, Before campaign	“People prefer oral vaccines, because from what I heard, people believe that injections bring viruses, that you would easily contract the virus.”
11	Man 2 doses, after campaign	“I have heard that people have wrong information. You find that some women will abort the child sometime after taking the vaccine and people will say, “It is because of the vaccine; that is why the unborn baby has died.”
12	NHC member, after campaign	During the second dose [vaccination campaign], we found a woman with her divorced daughter who claimed to have suffered a miscarriage after taking the first dose. The mother then told us that her daughter cannot go for the second dose because it is not safe.”

### 3.3 Competing beliefs about vaccination

Some residents in urban areas preferred informal, traditional and religious approaches to prevention and cure. Participants described cases of young men using beer, spirits and local alcohol, *Tujilijili, Junta,* and *Kachasu*, while others used other informal and traditional alternatives such as traditional brews, herbs and tattoos (Table 4, quotes 1 & 2). Some people avoided vaccination based on religious grounds including religious explanations *(“God did not take any medicine”)* and association of vaccines with Satanism (Table 4, quotes 3 & 4). In particular, HCWs described some churches that promoted faith healing eschewing modern medicine:

*“Some churches are difficult, (…) they say; “it [vaccines] is a sin to God. God heals everyone (…) Me, I pray, my God will heal me.”*

NHC member, before campaign

Furthermore as shown in Table 4, quotes 5 & 6, the association of vaccines with “*white*” or “*western*” medicine also created suspicion that *“whites don’t get vaccinated,*” and only Africans were targeted due to ulterior motives such as *“experiments to see if [vaccines] will work,”* killing or sterilizing people:

*“(*…*) We have never seen on TV, or heard on the radio, showing the vaccine being given to people in western countries. All we see is here in Africa, (*…*) We have never seen queue of people being vaccinated in Asia, Europe or America (*…*)”*

NHC member, after campaign

### 3.4 Previous experiences with vaccines

Some discussants knew people who refused vaccination because they, their family or friends became “ill” soon after. Examples included nausea and vomiting after OCV, “rash” and “stomach pains” after measles vaccination and “high temperature” and “rash” after Rubella vaccination. As illustrated in Table 4, quotes 7 & 8, news of side-effects from informal sources fuelled vaccine hesitancy.

Among respondents supporting the use of oral vaccines over injections, a few mentioned fears of being injected incorrectly or contracting infections (Table 4, quotes 9 & 10), but most agreed that people were afraid of pain:

*“So the reason why people don’t like injections is because they just have that fear that injections are painful. Others when you ask, the last time they were injected is a long time, when they were may be 7 years old. And from that time, they never injected because of fear.”*

Man, one dose, before campaign

Also, though most pregnant women were thought to be receptive to vaccinations, some reportedly refused vaccination for fear of harm to their unborn child particularly if the decision to abstain from vaccination was supported by elders (Table 4, quotes 11 & 12). Some elders who thought vaccines were unnecessary also influenced young people not to vaccinate their children:

*“(*…*) when you are staying with grandmother, if it is time to take the baby for injection, they refuse saying that, “These injections! From the time I grew up I have never done that to my children!”*

Woman with one dose, before campaign

### 3.5 Misconceptions and perceived effectiveness

As presented in Table 5, perceptions of vaccine effectiveness were often grounded in misconceptions about how, for whom and for how long vaccines work. Respondents believed that vaccines worked against illnesses (Table 5, quotes 1 and 2), particularly for childhood illness, rather than being disease-specific. They observed how measles and polio became less prevalent after the vaccine was introduced:

*“I think if they hear that they are vaccinating children, most of them feel good because [vaccines] protect children. There are very few children who would be found sick of polio these days.”*

Woman, two doses, after campaign

**Table 5:** Misconceptions and perceived effectiveness of vaccines among laypersons and health actors.

**Theme: Misconceptions and Perceived Effectiveness**		
**Sub-Theme:** Vaccines are perceived to be effective		
1	Man, 2 doses, after campaign	“I take them seriously myself because they [vaccines] protect us from a lot of diseases. For instance, when we took the cholera vaccine, automatically I was 100% sure I will not get sick from cholera. Then, there is the example of polio, when you have some young child, a baby vaccinated against polio, you are 100% sure to say he/she won’t get polio.”
2	Lay HCW, after campaign	“Then the other thing that they think is that, the moment you are vaccinated you can play around anyhow, anywhere. That if you’re vaccinated, you cannot get sick. ”

**Sub-Theme:** Name, mechanism, purpose and duration of cover differs by vaccines is not well understood		
3	Woman 0 doses, after campaign	“Injection work faster than the one for oral because once they inject you, it starts to work.”
4	Woman, 1 dose, after campaign	“Others do not think about these vaccines we have been given, how is it going to protect us in future? For example, the vaccine for tetanus, you need to be injected five times. You find that maybe you were given the vaccine once; you refuse to be vaccinated again without knowing why they are giving you the vaccine again.”
5	NHC member, after campaign	“Some people may have a virus in their body. Then after taking the vaccine, you will find that the disease will now show itself. This person will get sick but will not die. ”

As shown in Table 5, quote 3, some residents incorrectly thought that injections were more effective as they worked faster than oral vaccines:

*“It is better to be given injections than oral medication, because an injection goes direct to the blood stream. But the one for swallowing requires to dissolve, be digested for it to start working in the body.”*

Man, one dose, before campaign

Certain discussions suggested that some people incorrectly believed vaccines provide overall rather than disease-specific protection as illustrated in Table 2, sub-theme ‘Vaccines are perceived to be effective’ and in the quote below:

*“I think they work. When I remember back then, before children were vaccinated against polio and measles, we were experiencing a lot of children with chicken pox. But now since vaccines came, it has reduced.”*

Man with one dose, after campaign

Furthermore, while residents correctly named different vaccines provided in their communities, some mentioned other products, such as Vitamin A, antiretrovirals and malaria medication. The quote in Table 2 under sub-theme ‘Name, mechanism, purpose and duration of cover differs by vaccines is not so well understood’ provides an example of conflating vaccination with other initiatives. This conflation of other initiatives with vaccinations was compounded by lack of reference to protection period when talking about vaccines (Table 5, quotes 4 and 5). When prompted with regards to OCV, respondents reported different durations with many acknowledging ignorance in this area.

### 3.6 Preferences for vaccine delivery

As shown in Table 6, laypersons were very keen to learn more about vaccines. Discussants emphasized they wanted more *“education about the vaccines”* because they’re *“just told that you should come for the vaccines at the clinic”* without being informed on what to expect in this area:

*“lf people are taught, they have no fear because they are knowledgeable about it. They would already have known the side effects, for e.g., you will vomit or have rash. Like some people who were vaccinated [took OCV] last time, you would hear them saying that “With me, I vomited or had rash.” So they [the organizers] need to start earlier and tell them that once you take this vaccine you may vomit.”*

Man with zero doses, before campaign

**Table 6:** Preference for vaccine delivery among laypersons and health actors.

**Theme: Preference for Vaccine Delivery**		
**Sub-Theme:** There is desire for more, and more accurate information from trusted sources		
1	Woman, 2 doses, after campaign	“Volunteers can suit the person because people would feel shy of the health worker. But if it is a volunteer, we know each other here in the compound. You tell them everything, even the vaccine, if you want it or not. Then they will explain to you and convince you till you take part.”
2	Vaccinator, after campaign	“We feel that these vaccines are safe especially if those that sensitize have gone around to explain to people how it works. Because if they don’t explain, everyone is scared and asks, “How come you people at the clinic are getting vaccinated?” But if they go around and explain and if people know how the medicine works, people will not question vaccines.”

**Sub-Theme:** People want better access to vaccinations		
4	Woman 0 doses, after campaign	“Door to door helps those that cannot go to the clinic, like those with disabilities.”
5	Lay HCW, before campaign	“Mobile campaigns are very good because other people can’t manage to move long distances (…) People find difficulties to go to the clinics.”
6	Vaccinator, before campaign	“Another challenge was how [vaccines] to give the aged who stay very far. Their grandchildren would come to ask for the vaccine to give them.”
7	Woman 2 doses, after campaign	“Routine is better because all we need is a reminder. We need to hear that message every day. if [the message] comes like in a campaign, we forget. Maybe you are not there on the day of the campaign, but if it’s every day, you will know. It will just be clinking the mind that I need to do this.”
8	LHCW, after campaign	“There are those who prefer routine [vaccination]. We remember when we were doing the cholera [vaccine campaign], there are those who were saying, “We still have time, we will go.” Then when they heard that it’s the last day, that is when they came in large numbers. So they prefer routine.”

Discussants had several recommendation on how to ensure reach and coverage. They supported vaccine delivery by both HCWs and volunteers. HCWs were considered knowledgeable but busy and, as shown in Table 6, quotes 1 and 2, volunteers were thought to be better at reaching people in the community as they belonged to it:

“*The volunteers are suitable because these people live in the community. They live together, eat together, and [people] are used to them. It’s very easy to understand a person who you live with*.”

Woman with one dose, after campaign

They also emphasized the importance of including influential leaders and elders during campaigns:

“*In future campaigns, I think it is also important to involve churches because different churches have different beliefs. The community are involved with the pastor, the headmen, and the bishop. If we educate those people, even once you talk about like VCT [Voluntary Counselling and Testing], these days, pastors talk about it and the elders talk about it. People are encouraged because those are the people they tr*ust.”

Vaccinator, before campaign

As illustrated in Table 6, quotes 4–6, people were concerned about reaching community members not willing or able due to limited mobility to go to health facilities for vaccinations, leading to the suggestion that mobile campaigns may be more effective in raising awareness and uptake:

*“Door to door is good because others can’t walk because they are sick. It would help if you followed them to their door steps. People want [to be vaccinated], but don’t have means of moving*.”

Male, two doses, after campaign

Mobile campaigns aligned better with people’s routines, allowing them to take a vaccine without giving up other responsibilities:

*“There and then you give them [vaccination], they will take [it], unlike when a person is on business thinking, “I leave here, I go to line up there, what time will I leave that place? My business will do what?! [It]will suffer.”*

NHC member, before campaign

Similarly, Saturdays and Sundays were the preferred days for a vaccination campaign, due to fewer work and social commitments:

*“Saturday because most people don’t work or work half-day and sunday because when people are back from church they find time to go to the clinic.”*

Man with zero doses, after campaign

*“Most of those who never got the vaccine were the men because they go for work early in the morning and by the time they knock off they would find that we have stopped giving the vaccine. They [Ministry of Health] should include days like Saturday so that most of the people should benefit.”*

Vaccinator, before campaign

Laypersons had mixed preferences for routine and campaign-based delivery (Table 6, quotes 4–8). Routine delivery allowed information to percolate, while campaigns raised attention due to their intensity and motivated action. However, several respondents did not understand the nature of each type of delivery, believing, for example, that routine campaigns would require several visits irrespective of type of vaccine.

## 4 Discussion

While respondents agreed that vaccines were perceived as acceptable, safe and effective, they also described how traditional and religious alternatives, past experiences with interventions, social norms and interactions, and incomplete understanding and access to vaccination interacted to foster hesitancy in their communities. Responses from health actors were acutely in tune with those in the community, likely because they are from the community and not professionalised.

### 4.1 Acceptability and perceived safety

#### 4.1.1 Competing beliefs about vaccination

In our study, we found acceptability and perceived safety enmeshed with competing traditional and religious beliefs and distrust of modern medicine rooted in history. Normative use of traditional remedies is well-established in the country and coexists with modern medicine [20], [21], with people commonly relying on hospitals for physical afflictions [21], [22]. The use of alcohol for prevention of illnesses was not described as generalized in the community, but the reportedly prevalent use by young men deserves more study. Use of prayer and traditional remedies could be fuelled by distrust of modern medicine which is still perceived as “*white*” or *“western”* [20], [21]. This distrust finds its roots in colonial history of exploitation and appropriation [21], [23]. In the last decades, rumours and lack of knowledge around biomedical research [23], [24], [25] have fuelled the distrust as has been observed in Zambia for HPV vaccine [9]. Rumours of links between western medicine and Satanism have been also described in the city recently [14]. In this context, traditional healers might be seen as more familiar local alternatives [21]. Similarly, faith healers, who espouse love, reconciliation and the “power of God”, and cast other choices as being the work of ‘Satan’, can be more convincing [20], [21]. Reports of some churches discouraging vaccination are of special concern, given their importance in the diffusion of health messages and raising awareness towards vaccination in Zambia [26].

#### 4.1.2 Previous experiences with vaccines

In our study, family and friends’ experiences with vaccines influenced the decision to be vaccinated both in positive and negative ways. Perceived reductions in the number of childhood diseases drove generalized approval of vaccination; however, stories about adverse effects coupled with paucity of information about them could be responsible for pockets of refusal. Past personal and community experiences with vaccination are known to define public opinion towards vaccines [3], [7]. Negative experiences of adverse effects, worsened by limited knowledge, have been reported as barriers to vaccination in the city [14]. The same study reported that participants generally thought it acceptable to be vaccinated during pregnancy. In contrast, our study found some existing fears for the foetus, the newborn, and mothers’ fertility. As found by two other studies in Zambia [9], [14], elders and community leaders may play a bigger role than health care workers in influencing the decisions of younger family and community members.

### 4.2 Misconceptions and perceived effectiveness

Our study found that incorrect expectations of vaccine protection were present in the community. Just as adverse events influenced perceptions on safety, successful vaccination programmes such as polio might have fostered expectations of life-long and generalized protection. Similar situations have been described in India, Philippines and Indonesia, where some community members believed vaccines contributed to overall improvent of children’s health and protection against disease rather than disease-specific focus, probably as a result of limited knowledge and vague messaging about vaccines [13]. Limited knowledge on vaccines and overlap between cure and prevention have been recently identified across mothers in Lusaka [14] and appears to persist in our study population. Our local colleagues pointed out that in Lusaka, where Nyanja is more colloquial than formal, “*Mankwala*” is the synonym for vaccine and medicine. In Bemba language, vaccine is called “*umuti uwakuichingilila”* (medicine for protection), which could easily have respondents believing in medicines as prevention and vaccines as treatment and might have generated unrealistic expectations on effectiveness. These expectations can act as a precursor of vaccine hesitancy if immunized individuals start becoming infected [3], [13]. In such a scenario, distrust can go beyond vaccination and, combined with existing rumours and concerns, compromise trust relationships with providers and the health system [3], [27].

### 4.3 Preferences for vaccine delivery

Community’s suggestions and preferences for increasing the perceived importance and convenience of being vaccinated were focused on improving vaccine literacy and access to vaccines among community members. Interestingly, these community-led recommendations align with evidence from a contemporaneous study reporting that limited understanding of vaccines was driving vaccine hesitancy and demand for education [14]. They also align with recommendations on community-based education in urban poor communities [18], [28] and dialogue-based interventions and social mobilization [29] as successful approaches to increase demand for vaccination.

To improve access, respondents also supported initiatives such as mobile, door-to-door campaigns and weekend delivery, addressing well known barriers to access, including long working hours, distance to health facilities and limited physical mobility of some community members [3], [10], [18], [30]. These initiatives align with evidence that outreach services increase immunisation coverage in children by reducing the distance between a household and a vaccine-supply point in low income peri-urban areas [10], [11]. Similarly, communities’ interest in having volunteers to support vaccination campaigns is in line with lay HCWs’ adequacy in promoting immunization [31]. This feedback establishes a set of potential interventions backed by both evidence and the community that should be considered to address access-related issues in this setting.

### 4.4 Addressing sources of hesitancy and improving access

Our study reveals the complexity of factors related to confidence, complacency and convenience that fuel vaccine hesitancy [4] in peri-urban areas in Lusaka. Participants in our study perceived vaccines as effective and the health system as trustworthy with knowledgeable HCWs and approachable volunteers. However, this confidence [4] lies on shaky foundations of meaning embedded in the intertwined terms ‘medicine/vaccine’ and ‘prevention/treatment, past experience, and vague messaging. In the absence of vaccine-specific knowledge, participants reported that news of side-effects and rumours rooted in past experiences [20], [21], [22], [23], [24], [25] made donors’ and policy-makers’ motives suspicious. Also, while our study population demonstrated both concern regarding illnesses and belief in vaccine efficacy as evidenced by disease reduction, competing beliefs in traditional and religious practices as protective agents promoted complacency [4] in some of our study population. In poor peri-urban areas such as our study area, complacency was further rooted in the inability of adults to reprioritize other caregiving and income-generating activities to seek vaccinations for themselves and their children. For example, many did not think they could transport the aged and differently abled or get leave from work. Thus, even when made convenient [4] through mobile campaigns and free of charge provision, low perceived self-efficacy, low geographical access and low literacy were reported as affecting uptake. Participants’ suggestions to increase the time and place of vaccine availability aim to increase convenience. However, an increase in this aspect of convenience needs to be accompanied by improved utilization of influential social networks, norms of communication, and cultural terminologies and concepts of prevention and treatment, to counter competing narratives that delay or stop vaccine uptake [12].

To maximize uptake, educational and vaccination campaigns should align with communities’ preferences for delivery on weekend, door-to-door and by volunteers from the same communities. Educational and engagement efforts should target key influencers such as elders and religious leaders and also be embedded in services for key groups such as pregnant women [32], [33]. Based on our research, education should provide information on vaccines’ aim and possible adverse effects, to limit the spread of rumours and distrust. Furthermore, to address misconceptions about vaccine effectiveness and prevent future frustration leading to vaccine hesitancy, vaccine-related education should be pre-emptive, differentiate between vaccines and other medical products using clear language, and be clear with regards to the effectiveness of different vaccines [33]. Increased transparency and information to communities through trained community advisory boards selected from the same community also seems a promising approach to improve understanding [34].

## 5 Limitations

Given the nature of this study, prevalence of these determinants of hesitancy cannot be quantified. Moreover, given our sampling method and connection to the OCV campaign, people living in poorly accessible areas or distrusting vaccines might be underrepresented. People with poorer access to vaccine provision and awareness-raising interventions—who might have lower education, be living in remote areas in the compounds or be newcomers from rural areas—are likely to be more influenced by some determinants of hesitancy and less represented in our sample [10], [18]. Finally, the use of focus groups prevented us from collecting sensitive information such as HIV status, previously correlated with incomplete child immunization in the country [35]. On the other hand, the structure of the FGDs, with a minimum number of people with zero doses required, aimed to balance the representation of individuals less keen to vaccinate and led to very specific recommendations to address vaccine hesitancy.

## 6 Conclusion

Vaccine-hesitant individuals are likely to be influenced by past experiences with vaccinations, fear of injections, distrust of western medicine, competing belief models based on traditional and religious practices and barriers to access. Limited understanding of vaccines’, including an overlap of medical concepts in local languages are likely to contributors to incorrect beliefs on vaccines effectiveness and duration that could lead to future distrust of vaccines and providers. Discussants’ preferences for delivery were generally in line with evidence and addressed the problems identified in the community. The need for further community education and transparency on vaccines was identified as paramount to reduce hesitancy, as well as engaging and educating key influencers such as elders and religious leaders. Access could be improved through mobile campaigns that combine healthcare workers with volunteers from the community, adapt to community schedules and target individuals with reduced access. We recommend further research to understand how lack of sufficient information drives vaccine hesitancy, identify whether rumours and competing beliefs affect particular groups and what are the most effective ways of providing education and improving access.

## Conflict of interest

Authors report no conflict of interest.

This study was funded by the Bill & Melinda Gates Foundation (BMGF), through the Agence de Médecine Préventive (AMP) and the VaxiChol Project.

## Author contributions

MPG, LH, AS developed the study protocol and guides with inputs from EG, RD and RC. LH, MPG, CM, and SN analysed the data on NVivo and synthesized it along with AS. MPG was the main author, all authors contributed to the first draft; AS, LH and RC made critical revisions. All authors have given final approval of the manuscript.
